# PVA-TiO_2_ Nanocomposite Hydrogel as Immobilization Carrier for Gas-to-Liquid Wastewater Treatment

**DOI:** 10.3390/nano14030249

**Published:** 2024-01-23

**Authors:** Riham Surkatti, Mark C. M. van Loosdrecht, Ibnelwaleed A. Hussein, Muftah H. El-Naas

**Affiliations:** 1 Gas Processing Center, Qatar University, Doha 2713, Qatar; 2 Department of Biotechnology, Delft University of Technology, 2628 CD Delft, The Netherlands

**Keywords:** polyvinyl alcohol (PVA), nano-gel, porosity, compression strength, biomass, water purification

## Abstract

This study investigates the development of polyvinyl alcohol (PVA) gel matrices for biomass immobilization in wastewater treatment. The PVA hydrogels were prepared through a freezing–thawing (F-T) cross-linking process and reinforced with high surface area nanoparticles to improve their mechanical stability and porosity. The PVA/nanocomposite hydrogels were prepared using two different nanoparticle materials: iron oxide (Fe_3_O_2_) and titanium oxide (TiO_2_). The effects of the metal oxide nanoparticle type and content on the pore structure, hydrogel bonding, and mechanical and viscoelastic properties of the cross-linked hydrogel composites were investigated. The most durable PVA/nanoparticles matrix was then tested in the bioreactor for the biological treatment of wastewater. Morphological analysis showed that the reinforcement of PVA gel with Fe_2_O_3_ and TiO_2_ nanoparticles resulted in a compact nanocomposite hydrogel with regular pore distribution. The FTIR analysis highlighted the formation of bonds between nanoparticles and hydrogel, which caused more interaction within the polymeric matrix. Furthermore, the mechanical strength and Young’s modulus of the hydrogel composites were found to depend on the type and content of the nanoparticles. The most remarkable improvement in the mechanical strength of the PVA/nanoparticles composites was obtained by incorporating 0.1 wt% TiO_2_ and 1.0 wt% Fe_2_O_3_ nanoparticles. However, TiO_2_ showed more influence on the mechanical strength, with more than 900% improvement in Young’s modulus for TiO_2_-reinforced PVA hydrogel. Furthermore, incorporating TiO_2_ nanoparticles enhanced hydrogel stability but did not affect the biodegradation of organic pollutants in wastewater. These results suggest that the PVA-TiO_2_ hydrogel has the potential to be used as an effective carrier for biomass immobilization and wastewater treatment.

## 1. Introduction

Biological treatment is a common and eco-friendly way to clean wastewater. It uses tiny living organisms to break down pollutants. This natural approach is crucial for sustainable wastewater management, contributing to environmental protection and the preservation of water quality [1]. There are two main types: free and immobilized systems. In free systems, these tiny organisms float in the wastewater, freely moving around and cleaning it by interacting with pollutants [2].

Compared to free biomass, immobilized cells have several advantages, including reducing the reactor volume, enhancing the treatment stability, and increasing the biodegradation rate and biomass growth. These advantages are attributed to the large protected surface area provided for the biomass to grow when the carriers are suspended in water [3,4].

Several natural and synthetic polymers have been used for biomass immobilization. Polyvinyl alcohol (PVA) is a promising polymer type that is widely used in the areas of biotechnology and wastewater treatment. It is inexpensive, non-toxic, and has high chemical and mechanical stability [5]. PVA gel can be prepared using physical or chemical cross-linking methods. Chemical cross-linking requires the addition of chemicals, such as boric acid and alginates, to complete the cross-linking process, whereas physical cross-linking involves thermal methods using iterative freezing–thawing (F-T) cycles. Compared to chemical cross-linking methods, physical cross-linking is safer for microorganisms, and the resulting gel has higher mechanical strength [6]. The preparation of PVA gel using physical cross-linking through freezing–thawing (F-T) cycles depends on two parameters. The first is the formation of a network, which binds a large number of water molecules together. The second is a strong chain interaction to form semi-permanent joints in the molecular network [7]. The PVA hydrogel forms a three-dimensional structure that does not dissolve or absorb water but can swell in the aqueous solution. The properties of the PVA gel prepared by F-T cycles depend on the polymer weight % in aqueous PVA solution, the time and temperature of freezing and thawing, and the number of F-T cycles [8,9]. Thus, the properties of the PVA gel differ according to the preparation conditions.

Generally, PVA gel has high mechanical strength and a porous structure. The importance of the porous structure is to allow the entrapment of biomass inside the polymeric matrix, while facilitating mass transfer of the organic contaminants into the gel. The hydrogel is synthesized around the biomass and enclosed in the porous polymeric matrix, which allows the diffusion of nutrients, substrate, and products [10,11]. The porous structure of polymeric carriers would facilitate the entry of substrates and pollutants, and hence enable the microbial degradation of pollutants in wastewater [12].

Historically, composite materials were primarily employed in the manufacturing process to enhance strength and provide reinforcement. This conventional application aimed to capitalize on the combined properties of different materials. However, a contemporary approach has emerged that responds to specific challenges in various industries. This involves the integration of nanostructured fillers into hydrogels, a technique that has gained popularity for creating innovative materials with multifaceted functionalities. This recent trend signifies a shift from traditional strength-focused uses of composites towards a more nuanced and versatile utilization, allowing for tailored solutions to address diverse industrial demands [13]. This includes the addition of organic, inorganic materials and the incorporation of nanoparticles in PVA hydrogel matrices to produce nanocomposite hydrogels [14]. These additives resulted in modified hydrogels with good mechanical strength, a large surface area, and a controllable pore structure [14]. Generally, the addition of the nanoparticles in the polymeric composites resulted in a new polymer matrix with unexpected properties that significantly differ from the conventional materials. Several nanoparticles, such as gold (Au), silver (Ag) iron oxide (Fe_3_O_4_, Fe_2_O_3_), alumina (Al_2_O_3_), titanium (TiO_2_), and zirconia (ZrO_2_), were combined with PVA gel to form PVA/nanoparticles hydrogel composites for an extensive range of biomedical applications, including drug delivery and artificial tissue engineering [15,16]. However, the amount and the type of nanoparticles are the most critical factors in obtaining nanocomposite polymers with high mechanical strength [17].

The use of TiO_2_ to reinforce hydrogels has been studied in numerous investigations utilizing PVA gel with other materials. They showed that TiO_2_ was used for enhancing the mechanical strength and pore structure of the hydrogels. It was proposed that mixing of TiO_2_ nanoparticles with a biopolymer consisting of PVA and iota-carrageenan (CRG) prepared by F-T cycles resulted in a stable hydrogel with improved mechanical strength with an approximately 20% increase in Young’s modulus by adding 0.25% TiO_2_ compared to the pure hydrogel [18]. Recently, Wang et al. concluded that adding dimethyl sulfoxide (DMSO), and TiO_2_ to PVA hydrogel composites enhanced mechanical strength, which resulted in 99% improvement in tensile strength by adding 0.3% TiO_2_ and 60% DMSO to 15% PVA gel. Nevertheless, in the above research, the performance of the PVA/TiO_2_ hydrogel was not studied without the addition of other chemicals during hydrogel preparation [19]. In addition, several studies examined the effect of iron oxide nanoparticles on mechanical characterization prepared by a simple F-T technique. Most previous studies highlighted the effect of iron oxide on PVA gel properties at high iron oxide (wt%) content in terms of their magnetic properties. For example, Hou et al. [20] concluded that the reinforcement of 10 wt% PVA with 10% iron oxide prepared by F-T cycles resulted in an 80% improvement in compression strength.

Although the application of PVA/nanoparticles such as Fe_2_O_3_ and TiO_2_ has been investigated in several areas, including biomedical applications, there are still limitations on applying physically cross-linked PVA/metal oxide nanoparticle composites for biomass immobilization. Additionally, no previous studies have compared different nanoparticles as reinforcement materials to enhance hydrogel properties. Thus, this work aims to study the reinforcement of PVA gel with metal oxide nanoparticles, such as Fe_2_O_3_ and TiO_2_ for biomass immobilization. The work involved the development of PVA/metal oxide nanoparticles with 10 wt% PVA with the addition of a small content of nanoparticles ranging from 0.02 to 1.0 wt%. Furthermore, the study investigated the influence of nanoparticle type and content on the morphology and mechanical properties of the PVA/nanoparticles hydrogel composites. The prepared PVA gel composites were then tested in a bioreactor and compared with the pure PVA gel to understand the immobilization matrix’s performance and stability.

## 2. Materials and Methods

### 2.1. Materials

Polyvinyl alcohol (PVA) powder of analytical grade was obtained from BDH, Manchester, UK. All nanoparticles, including iron oxide (Fe_2_O_3_) and titanium oxide (TiO_2_) with particle sizes ranging from 50–200 nm, were obtained from Sigma Aldrich, St. Louis, MO, USA. All other salts for the preparation of the nutrient, such as MgSO_4_-7H_2_O, K_2_HPO_4_, CaCl_2_-2H_2_O, (NH_4_)_2_CO_3_, FeSO_4_-7H_2_O, ZnSO_4_-7H_2_O MnCl_2_-4H_2_O, CuSO_4_-5H_2_O, and CoCl_2_-6H_2_O, Na_2_MoO_4_-2H_2_O, were also obtained from Sigma Aldrich. Additionally, the COD reagent was obtained from HAC Company for the COD analysis.

### 2.2. Experimental Procedure

#### 2.2.1. PVA Gel Nanocomposite Preparation

Polyvinyl alcohol (PVA) matrices were prepared through the iterative F-T method, as prepared in previous studies [4,21]. Several sets of PVA hydrogel were prepared at 4 F-T cycles, including pure PVA and PVA/nanoparticles hydrogels with different nanoparticle types (TiO_2_ and Fe_2_O_3_) and contents. A specific amount (0.04 0.06, 0.2, and 2 g) of each nanoparticle was added to 180 mL of distilled water to create a well-mixed suspension, followed by the addition of 20 g of PVA powder to the nanoparticle solution to prepare PVA/nanoparticles solution with 10 wt% PVA and nanoparticles wt% of 0.02, 0.06, 0.1, and 1.0 wt%. Each solution was heated at about 70–80 °C and PVA powder was added slowly to the hot nanoparticle solution with continuous mixing to make a well-mixed homogenous solution. The mixture was then cooled to room temperature, poured into special molds, and kept in a freezer for 24 h at −20 °C before transferring them to a refrigerator and allowing a thawing process for 5 h at +20 °C. The freezing–thawing process was repeated for four cycles to ensure good cross-linking. All PVA matrices prepared were characterized to determine the morphology, bond formation, and mechanical behavior.

#### 2.2.2. SEM Analysis

The morphology and microstructure of the prepared PVA matrices were examined using Scanning Electron Microscopy (SEM), Nova Nano SEM 450, which can provide high-quality images with high resolution. All samples were freeze-dried before the morphological test. The pore size distribution and the average pore size were then carried out using the ImageJ software Version 154 program for each sample.

#### 2.2.3. Bond Characterization Using FTIR

The characterization of the bond formation was investigated using Fourier Transform Infrared Spectroscopy (FTIR) for the pure PVA and compared to the cross-linked PVA/nanoparticles hydrogels. All bonds were analyzed using the Attenuated Total Reflectance (ATR) FTIR analyzer using Transmittance Mode. A thin layer of cross-linked PVA and PVA/nanoparticle hydrogel was prepared by the F-T cycles, dried at room temperature using tissue paper, and installed in the FTIR, and the pure PVA hydrogel was used as a standard in FTIR analysis. Samples were pressed to the thin plates, and the FTIR spectra of PVA hydrogels were determined by direct transmittance. The spectra were then recorded for each sample using a Perkin Elmer Spectrum One FTIR Spectrophotometer. All spectra were analyzed at a spectral resolution ranging from 40 to 4000 cm^−1^. The FTIR spectra were corrected to the baseline correction, and the major vibration bands were then associated with their chemical groups.

#### 2.2.4. Mechanical Properties

The role of nanoparticles in improving the mechanical properties and strength of the PVA matrix is an essential factor in this study. All prepared nanocomposite PVA gels were tested to determine their mechanical strength and compared with pure PVA gel. After preparing the PVA gel composites, the gel matrices were cut into cubes with nominal dimensions W × L × H of 16 mm × 17 mm × 18 mm. The compression tests were performed on a universal material testing machine (MTS) with a 50 kN load cell. All samples were tested at room temperature and a constant overhead speed of 25 mm/min until the sample reached ultimate failure (complete compression). Mechanical tests were carried out to evaluate the compression properties through stress-strain curves of the PVA matrices (compression strength and Young’s modulus).

#### 2.2.5. Rheological Characterization

The rheological characterization was carried out using an Anton Paar MCR302 Rheometer. The analysis was carried out using a 25 mm diameter parallel plate with a 1 mm gap. The dynamic viscoelastic measurement was conducted at a strain value of 1.0%, which ensured hydrogel deformation within the linear viscoelastic region. Viscoelastic behavior was measured in a frequency sweep range from 0.1 to 100 Hz. From this analysis, both the storage modulus (G′) and loss modulus (G″) of the PVA/nanoparticles gel composites were plotted as a function of frequency.

#### 2.2.6. Stability and Performance of PVA/Nanoparticles Hydrogel in Bioreactor

The prepared PVA gel and PVA/nanoparticles hydrogel were cut into 1 cm^3^ volume particles and then added to a 1 L spouted bed reactor described in a previous study [22]. The reactor was continuously aerated, and the mass was evaluated at several time intervals. Around 300 mL of PVA gel and PVA/gel nanoparticles composites hydrogel were added into two different 1 L volume reactors with continuous aeration.

Bacterial strains were isolated from GTL process water, as in the previous study [23], immobilized in PVA gel composites hydrogel, and used for GTL process water treatment. The industrial wastewater was obtained from a local GTL process water treatment plant and had a COD content of 1100 mg/L. The mineral nutrient was added to the GTL process water as follows: 300 mg/L MgSO_4_-7H_2_O, 250 mg/L K_2_HPO_4_, 150 mg/L CaCl_2_-2H_2_O, 120 mg/L (NH_4_)_2_CO_3_, 3.5 mg/L FeSO_4_-7H_2_O, 1.3 mg/L ZnSO_4_-7H_2_O, 0.13 mg/L MnCl_2_-4H_2_O, 0.018 mg/L CuSO_4_-5H_2_O, 0.015 mg/L CoCl_2_-6H_2_O and 0.013 mg/L Na_2_MoO_4_-2H_2_O. All experiments were carried out in a total volume of 1.0 L with immobilized bacteria in a PVA gel matrix that contained 30% of the total operation volume. The temperature and the pH of the solution were adjusted at 30 °C and 7.0, respectively; the optimum operation conditions were chosen according to previous studies [22,24]. It is worth noting that all experiments and measurements have been repeated twice, and average values have been reported. The average error ranged from 2 to 5%.

## 3. Results

### 3.1. SEM Analysis of PVA/Nanoparticles Gel Composites

**Figure 1:**
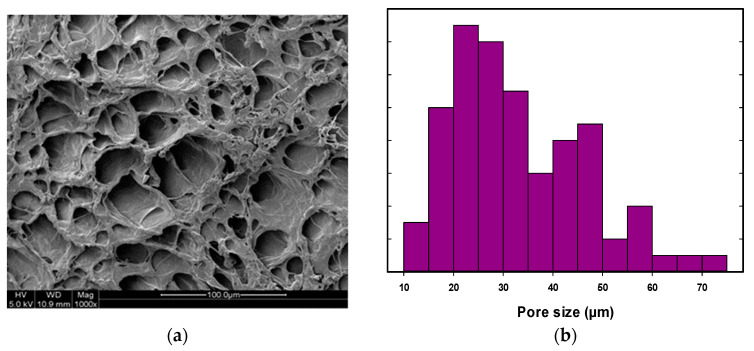
Pure PVA 10 wt% prepared by four cycles of F-T; (**a**) SEM image, (**b**) pore size distribution.

**Figure 2:**
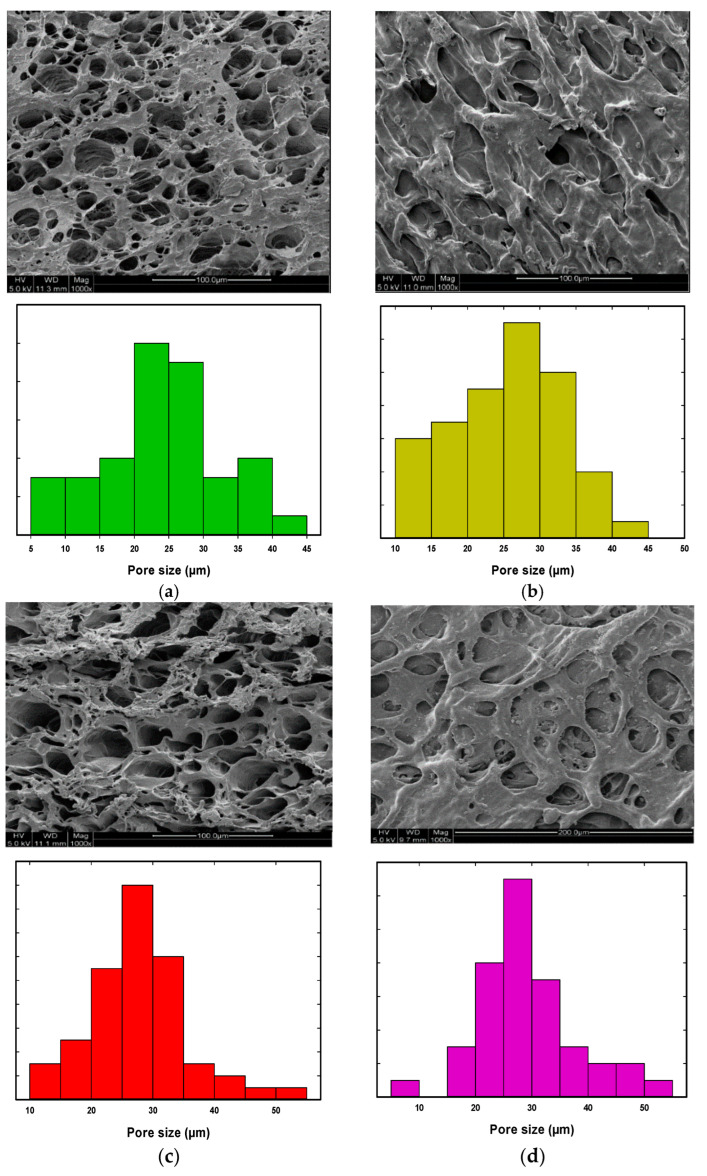
SEM analysis and pore size distribution of the PVA/Fe_2_O_3_ hydrogel; (**a**) PVA/0.02 wt% Fe_2_O_3_, (**b**) PVA/0.06 wt% Fe_2_O_3_, (**c**) PVA/0.1 wt% Fe_2_O_3_ and (**d**) PVA/1.0 wt% Fe_2_O_3_.

**Figure 3:**
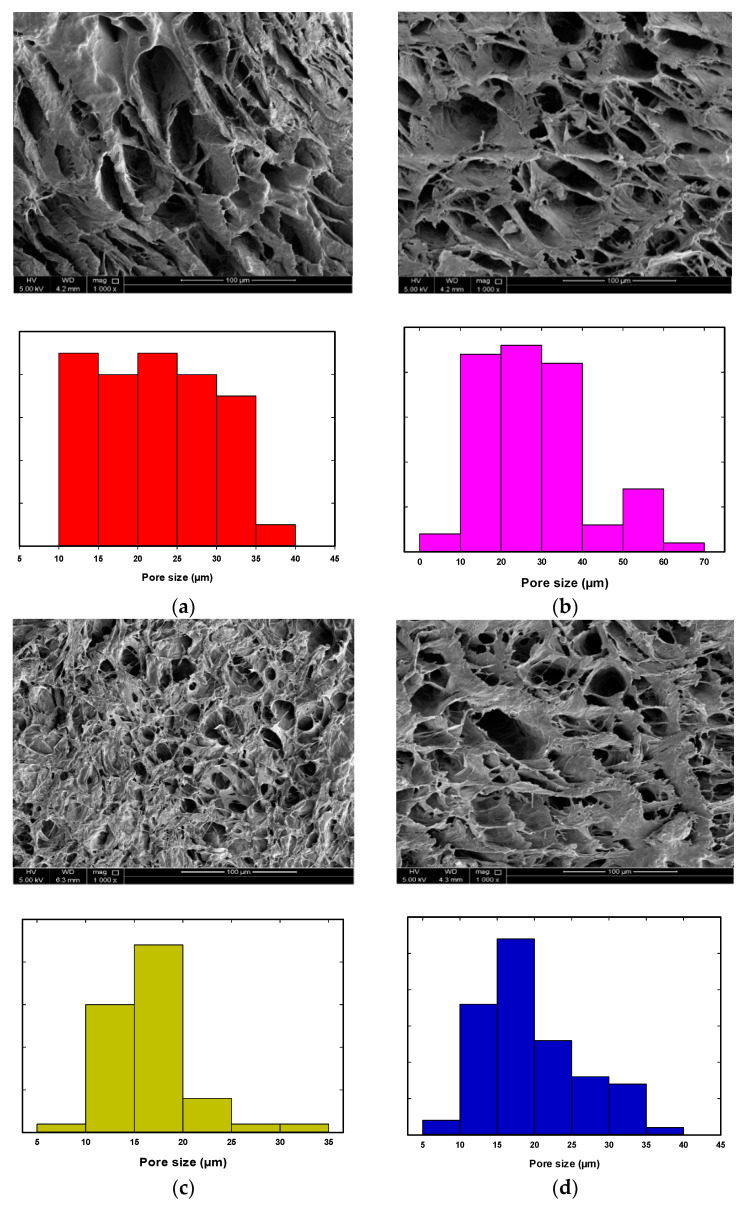
SEM analysis of the PVA/TiO_2_ hydrogel; (**a**) PVA/0.02 wt% TiO_2_, (**b**) PVA/0.06 wt% TiO_2_, (**c**) PVA/0.1 wt% TiO_2_ and (**d**) PVA/1.0 wt% TiO_2_.

The SEM images were obtained for all PVA hydrogel composites, and the pore size distribution was obtained for each sample using ImageJ analysis software. The SEM analysis and pore size distribution for pure PVA gel and PVA hydrogel composites with Fe_2_O_3_ and TiO_2_ at 0.02, 0.06, 0.1, and 1.0 wt% are shown in Figure 1, Figure 2 and Figure 3, respectively. All PVA hydrogel composites had a rough surface and a porous morphology with a sponge-like cross-sectional structure. As shown in Figure 1, the pure PVA gel showed a structure with a wide range of pore sizes ranging from 10 to 75 µm, and the average pore size was around 43 µm.

The SEM analysis of the PVA/TiO_2_ and PVA/Fe_2_O_3_ showed more heterogeneous pore size distribution within the polymer composites, and the addition of nanoparticles to the PVA gel resulted in more homogenous pores and a more regular pore’s structure. Additionally, an extra homogenous pore structure was observed compared to pure PVA gel (Figure 2 and Figure 3a–d). In the case of the iron oxide nanoparticles, the pore size decreased by increasing Fe_2_O_3_ nanoparticles from 0.02 to 1.0 wt%, and the average pore diameter was reduced from 32 to 25 µm. In contrast, the addition of small TiO_2_ contents (0.02 and 0.06 wt%) resulted in a large pore size, but the homogeneity of the addition of the hydrogel composites was enhanced. However, the addition of 0.1 and 1.0 wt% TiO_2_ reduced the pores’ size and enhanced the formation of a denser and more cross-linked hydrogel. Additionally, the pore size distribution of PVA/TiO_2_ gel resulted in an average pore size reduced to 20 μm and 35 μm by adding 0.1 and 1.0 wt% TiO_2_, respectively.

**Figure 4:**
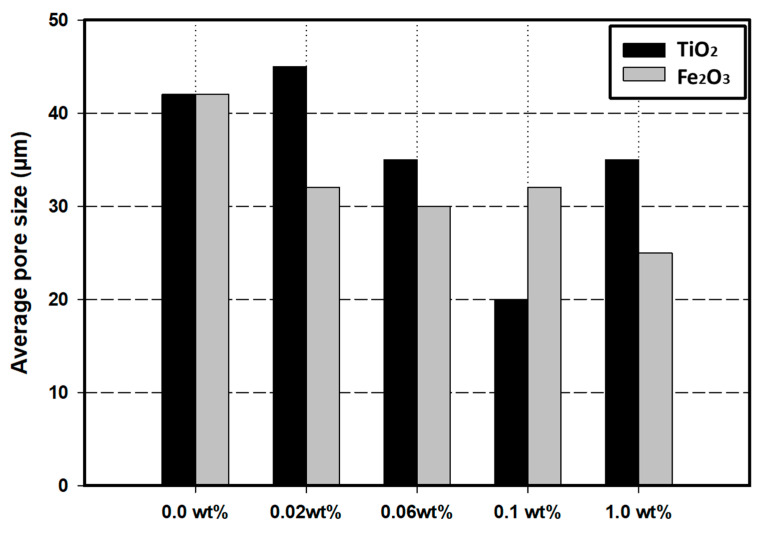
Average pore size for PVA/nanoparticles hydrogel at several nanoparticle contents.

The average pore size was obtained for the pure PVA gel and PVA/nanoparticles hydrogel composites. Figure 4 shows that the nanoparticles’ type and content had a significant influence on the PVA hydrogel composite pore size, and the average pore size noticeably decreased as the amount of nanoparticles increased. This can be explained by the presence of a mixture of polymer and nanoparticles, which decreases the possibility of the formation of large pore sizes. Moreover, the pore size distribution becomes more uniform by adding the nanoparticles than the pure PVA gel. Additionally, the pore size was decreased by increasing TiO_2_ wt% up to 0.1% (20 µm), further increase in the TiO_2_ content resulted in a negative impact on the diameter of the pores (34 µm), and resulted in larger pore size and less uniform pore size distribution. This may be attributed to the agglomeration of the TiO_2_ at a higher wt% that caused a reduction in the cross-linking effect using the nanoparticles. In the case of the reinforcement of the PVA hydrogel using Fe_2_O_3_, the pore diameter decreased with the increase of the iron oxide content, and the lowest pore size (25 µm) was obtained by adding 1.0 wt% Fe_2_O_3_. 

### 3.2. FTIR Spectroscopy

**Figure 5:**
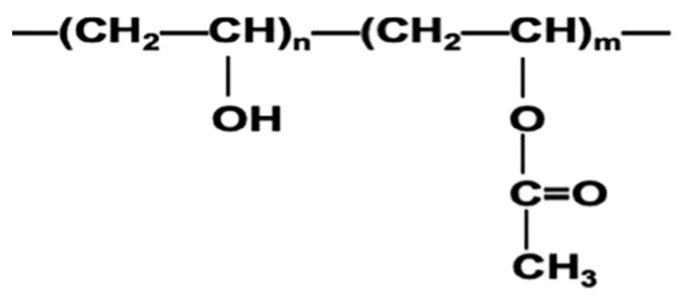
Chemical structure of PVA gel.

**Figure 6:**
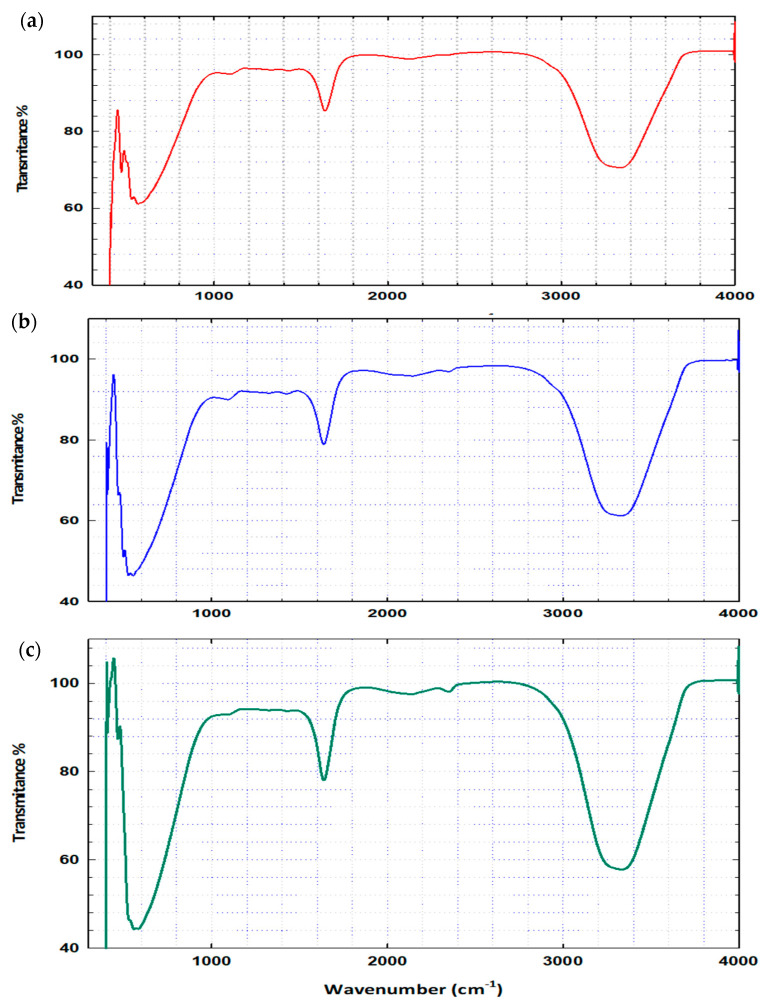
FTIR spectra of PVA hydrogel: (**a**) pure PVA, (**b**) PVA/Fe_2_O_3_ hydrogel, and (**c**) PVA/TiO_2_ hydrogel.

The hydrogel composition varies according to the polymerization monomer composition that is used in the hydrogel fabrication. However, the most general structure of the hydrogel consists of solvent, cross-linking monomer, backbend co-monomer, and electrolyte co-monomer [25]. The structure of the PVA gel is shown in Figure 5 [26]:

The FTIR spectra of the pure PVA (Figure 6a) show several bands that are distributed in the tested range of wavenumbers between 400 and 4000. Three distinct absorption bands appeared at 500, 560, and 590 cm^−1^, respectively. In addition, the bands observed at 1100 cm^−1^ is for the formation of the O-H bending and C-O stretching. The presence of the acetyl group (C-O) in the cross-linked PVA gel is usually present in the backbone of the PVA hydrogel. The vibration at 1690–1710 corresponds to the bending of the C=O stretching [27]. A strong broad peak is shown in the range of 3200–3400 cm^−1^, this is due to the presence of the O-H stretching obtained by the intermolecular bonding [28]. The FTIR spectra of the PVA/Fe_2_O_3_ hydrogel (Figure 6b) show bands at 450 and 500 cm^−1^, confirming the presence of iron oxide, which is usually characterized in this region [29]. In addition, the presence of the vibration 555 cm^−1^ confirmed the presence of the bond FeOC, which showed the bonding between the PVA gel and the Fe_2_O_3_ nanoparticles. For the PVA/TiO_2_ analysis several new peaks are shown. The peaks characterized as TiO_2_ are visible in the PVA-TiO_2_ hydrogel that suggests the interaction between TiO2 and PVA gel. As shown in Figure 6c, new spectra are observed in the region of 550 cm^−1^, that is generally characterized by the presence of the Ti-O-Ti group that usually appears in the range of 400 to 600 cm^−1^ [30]. Additionally, two more bands were observed at 440 cm^−1^ and 2350 cm^−1^ that may be assigned to the Ti-O-Ti stretching vibration and the stretching vibration of the O-H group to TiO_2_, respectively [31,32].

### 3.3. Rheological Analysis

**Figure 7:**
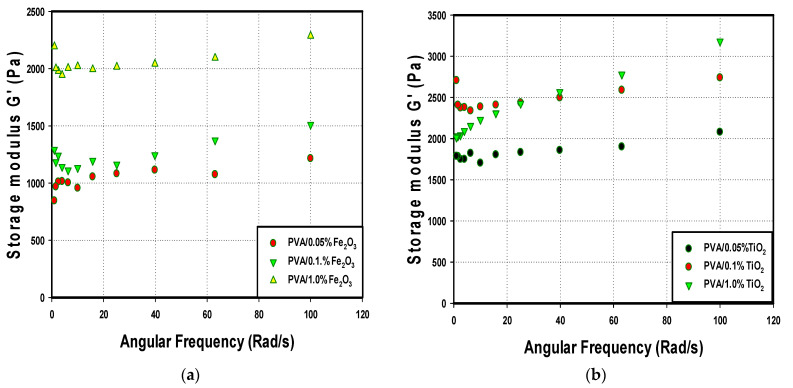
Frequency dependence of the dynamic storage modulus (G′) of the PVA/nanoparticles hydrogel (**a**) PVA/Fe_2_O_3_, (**b**) PVA/TiO_2_ hydrogels.

The elasticity behavior was investigated for the prepared PVA/nanoparticle composites, and the effects of the addition of the Fe_2_O_3_ and TiO_2_ composition of 0.05, 0.1, and 1 wt% on the storage modulus (G′) and loss modulus (G″) were evaluated. The linear viscoelastic frequency sweeps of several PVA/nanoparticles composites hydrogel are shown in Figure 7a,b. As observed, all hydrogels showed typical gel rheological behavior. Additionally, the storage modulus (G′) was larger than the loss modulus (G″) over the whole frequency range for all PVA hydrogel composites, showing that all hydrogel samples were highly elastic. The network structure of the PVA/nanoparticles composites hydrogel makes the hydrogels behave as an elastic material (G′ more than G″). The G′ values of all PVA/nanoparticles hydrogels increased as the frequency increased. However, the values of G′ were higher when the hydrogel was prepared by the addition of TiO_2_ nanoparticles, compared to Fe_2_O_3_.

For PVA/TiO_2_, the addition of TiO_2_ of 0.1% had a higher G′ than that at TiO_2_ of 1.0%. This suggested the formation of a new cross-linking network and increased network density. However, the addition of more TiO_2_ nanoparticles results in a negative effect on the mechanical strength of the hydrogel due to the agglomeration of TiO_2_ nanoparticles that has a direct impact on the cross-linking behavior of the gel. It was also demonstrated that the highest G′ value at zero angular frequency was obtained with the addition of 0.1 wt% TiO_2_. For the PVA/Fe_2_O_3_ hydrogel, G′ values increased with increasing Fe_2_O_3_ wt%, and the highest elasticity was obtained for the PVA/Fe_2_O_3_ with 1.0 wt%.

### 3.4. Mechanical Behavior

**Figure 8:**
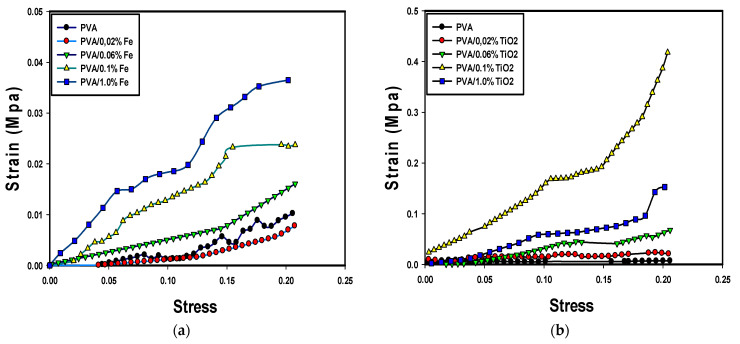
Stress-strain curve for the PVA nanocomposite hydrogel; (**a**) PVA/Fe_2_O_3_, (**b**) PVA/TiO_2_.

The compression strength of pure PVA gel was compared with PVA/Fe_2_O_3_ and PVA/TiO_2_ with nanocomposites at several contents of 0.02, 0.06, 0.1, and 1.0 wt% (Figure 8a,b). It should be mentioned that for the application of the PVA/nanoparticles composites in the moving bed bioreactor, the immobilization matrix will be exposed to low shear stress. Thus, the mechanical behavior was applied at a low range of strain (20%). The reinforcement of the PVA gel with nanoparticles improved the mechanical properties of the hydrogel. The stress-strain behavior of the hydrogel composites was compared with pure PVA gel. The stress-strain curves of several PVA/nanoparticles showed an improvement in the mechanical properties using both nanoparticles (Fe_2_O_3_ and TiO_2_), and the TiO_2_ nanoparticles resulted in an enhancement in the compression strength compared to Fe_2_O_3_. However, no improvement in the mechanical strength was observed at low Fe_2_O_3_ nanoparticles contents (0.02 and 0.04 wt%). In contrast, significant improvement in the mechanical strength resulted from increasing the Fe_2_O_3_ wt% at 0.1 and 1.0 Fe_2_O_3_ wt%. Clearly, the mechanical strength of the PVA gel composites was improved by the reinforcement of PVA hydrogel using TiO_2_ nanoparticles. The prepared PVA/TiO_2_ gel composite at 4 F-T cycles showed a high increase in the compression strength by adding TiO_2_ nanoparticles from 0.02 to 0.1 wt%, however, a negative impact on the mechanical strength was observed at higher TiO_2_ content (PVA/TiO_2_ 1.0 wt%).

**Figure 9:**
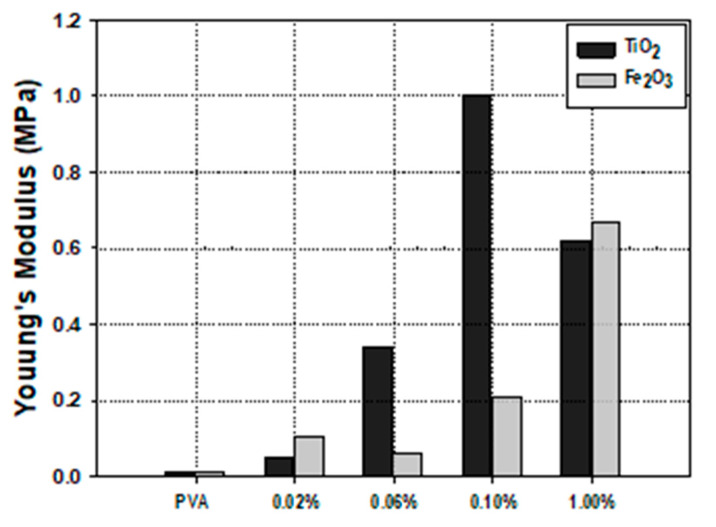
Young’s Modulus for PVA/nanoparticles hydrogel at several nanoparticle contents.

The effect of the type and content of metal-oxide nanoparticles on the Yonge’s modulus of the PVA hydrogel was illustrated. The prepared PVA hydrogel composites with metal oxide nanoparticles (Fe_2_O_3_ and TiO_2_) improved the mechanical strength of the PVA hydrogel composites (Figure 9). Clearly, the reinforcement of the PVA gel by TiO_2_ has a higher effect on Young’s modulus and mechanical strength. Maximum Young’s modulus (1.0 MPa) was obtained at TiO_2_ of 0.1 wt%. The results showed a 900% improvement in the hydrogel strength compared to the pure PVA gel. The Young’s modulus also increased with increasing the iron oxide content in the tested Fe_2_O_3_ wt% range. Compared to pure PVA, about 500% improvement was observed by adding 1.0 wt% iron oxide. Additionally, a 67% improvement in Young’s modulus was obtained in the PVA/TiO_2_ (0.1 wt%) compared to the PVA/Fe_2_O_3_ (1.0 wt%).

### 3.5. Stability and Biodegradation Performance Using PVA/Nanoparticles in a Bioreactor

**Figure 10:**
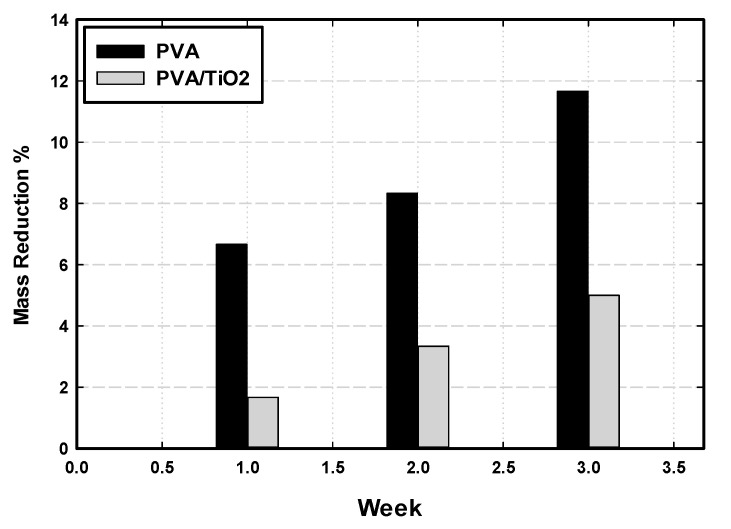
Mass loss in the PVA gel and PVA/TiO_2_ nanoparticles hydrogel in spouted bed reactor.

Among several PVA/nanoparticles hydrogel composites, PVA/TiO_2_ with 1.0 wt% nanoparticle composition showed high mechanical strength and stable porous structure. Thus, the PVA/TiO_2_ hydrogel’s stability and biodegradation performance were tested in a bioreactor and compared with the pure PVA gel. The durability of PVA/TiO_2_ nanoparticles hydrogel was compared with the pure PVA gel prepared by the F-T process through the loss in the PVA particles. The mass loss of the PVA hydrogel and PVA/TiO_2_ hydrogel composite were measured at different time intervals in two different spouted bed reactors operated under the same conditions (air flow rate 3 L_a_/L_r_. min and 1 L reactor volume). Figure 10 shows the mass loss over time over three weeks. PVA/TiO_2_ hydrogel composite is more stable and durable than the pure PVA gel, where around 50% improvement in the mass loss is obtained by using PVA/TiO_2_ hydrogel composite, compared with pure PVA.

**Figure 11:**
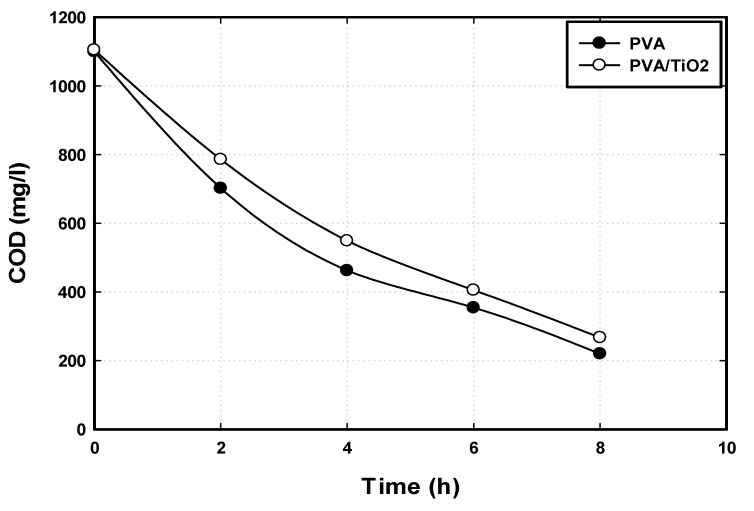
Biodegradation performance of immobilized bacteria in PVA and PVA/TiO_2_ hydrogel composites.

The biodegradation performance of the immobilized bacteria in the developed PVA/TiO_2_ hydrogel composites was compared with that of the immobilized bacteria in pure PVA gel. After one month of acclimatization, the biodegradation experiments were carried out using GTL process water with a COD of 1100 mg/L. The biodegradation performance was tested over 8 h in SBBR (Figure 11). The results showed that both immobilization matrices are suitable for bacterial growth and activity. Additionally, they resulted in high organic removal, with an insignificant difference in the biodegradation rate. Using immobilized bacteria in pure PVA and PVA/TiO_2_ hydrogel, the biodegradation rates were around 117 and 124 mg/L·h, respectively. Moreover, more than 77% COD removal was obtained using immobilized bacteria in both immobilization matrices. These results showed that the reinforcement of PVA gel with (0.1% wt%) TiO_2_ has no impact on the bacterial activity and bioconversion of pollutants in the wastewater.

## 4. Discussion

### 4.1. Morphological Analysis of PVA/Nanoparticles Hydrogel Composites

The pore structure is an important factor that should be considered when preparing PVA hydrogel for biomass immobilization. Generally, the porous structure is affected by increasing the polymer concentration since more polymer results in a shorter distance between molecular chains and increases the potential for hydrogen bonding and crosslinking. The addition of filler materials such as nanoparticles may shorten the distance and cause denser PVA hydrogels, reducing the pore size.

Generally, PVA hydrogel prepared by freezing–thawing has a highly porous structure with a micro-porous distribution in the entire hydrogel surface [4]. However, the SEM analysis showed that the pure PVA has a wide range in pore size distribution. In this study, incorporating nanoparticles into the PVA gel resulted in a more uniform pore structure with a smaller pore size. However, the effect of the type and amount of nanoparticles differed. The pore diameter decreased from 42 µm for pure hydrogel to 20 µm and 25 µm by incorporating 0.1 wt% TiO_2_ and 1.0 wt% Fe_2_O_3_, respectively. The addition of nanoparticles, such as Fe_2_O_3_ or TiO_2_ makes a more vital interaction between the nanoparticles and the hydrogen bonding, resulting in regular pore distribution [33]. The FTIR analysis of the PVA/TiO_2_ and PVA/Fe_2_O_3_ hydrogel composites highlighted new bands between the PVA and nanoparticles that agreed with the improvement in the morphological structure of the PVA/nanoparticles hydrogel composites. Moreover, regular and small pore formation is related to the efficiency of PVA crystallization, where the presence of the NP acts as additional crosslinking joints and forms more PVA chains incorporating in the gel network and decreases the crystaline size zone hence the size of the pores [33,34].

### 4.2. Elastic Behavior and Mechanical Properties of PVA Hydrogel Composites

The mechanical behavior of the hydrogel reflects both the homogeneity state and the interaction between the hydrogel and filler that increases the interfacial force within the cross-linked hydrogel composite [35]. It should be mentioned that the preparation of hydrogel composites using the freezing–thawing technique is a sensitive process; thus, the variation in the tensile strength, compression strength, and Young’s modulus is different according to the PVA molecular weight, conditions of freezing–thawing, and the addition of other chemicals during hydrogel preparation. Therefore, in most studies, the mechanical strength is usually compared with pure PVA gel prepared under identical conditions.

Generally, the aspect of the synthesis of stable and homogenous PVA metal-oxide composites is the presence of OH groups in the PVA polymer that have a high affinity to bind with the oxides present in the metal oxide nanoparticles and allow the distribution of nanoparticles and consequently avoid the formation of large flocculates. The rheological behavior and the mechanical tests of the PVA hydrogel composites showed that the reinforcement of the PVA hydrogel by both Fe_2_O_3_ and TiO_2_ nanoparticles enhanced the hydrogels’ elasticity and mechanical strength due to the formation of new crosslinking network points between the PVA chains and nanoparticles. The PVA hydrogel’s reinforcement using metal oxide usually resulted in new bonds that enhanced the crosslinking and consequently improved the mechanical strength. However, the nanoparticles’ amount must be controlled to achieve the required characterization and mechanical strength. The addition of the Fe_2_O_3_ to PVA increased the elasticity and mechanical strength by increasing the nanoparticle content up to 1 wt%. Whereas TiO_2_ improved the elastic behavior of the hydrogel by increasing the nanoparticle content up to 0.1%, and a negative impact was obtained at higher TiO_2_ contents (1.0 wt%). TiO_2_ nanoparticles, even at low contents, have more influence on the mechanical strength of PVA gel than the Fe_2_O_3_. The addition of TiO_2_ to PVA hydrogel resulted in strong interaction between the reactive groups (OH) on the surface of the PVA gel molecules and TiO_2_ nanoparticles [36]. Li et al. demonstrated that the improvement in the mechanical strength of PVA by adding TiO_2_ nanoparticles is due to the excellent interaction between TiO_2_ nanoparticles and the organic polymer that was observed from their FTIR analysis [37], which agreed with the bonding in this study. At low nanoparticles TiO^2^ concentration, low interaction between nanoparticles and polymeric material is obtained due to the considerable distance between nanoparticle molecules. Increasing the TiO_2_ wt% resulted in a shorter distance and a more robust interaction between TiO_2_ and PVA matrix [38]. It should be mentioned that the strong adhesion between TiO_2_ and the PVA matrix and the finely dispersed TiO_2_ particles in the polymeric matrix is also responsible for significant reinforcement of the mechanical properties of nanocomposite [38]. Additionally, TiO_2_ improved the mechanical strength due to hydrogen bonding and O-Ti-O bonding between TiO_2_ and hydrogel, which enhanced the mechanical strength. However, the TiO_2_ nanoparticles tend to agglomerate after a specific value due to the shorter distance between the suspended particles [18]. Li et al. compared the mechanical strength of PVA/TiO_2_ hydrogel prepared by 4.0 mass% PVA and 1.6 wt% TiO_2_ with pure PVA hydrogel and showed that the reinforcement of the PVA hydrogel with TiO_2_ nanoparticles resulted in a 30% improvement in the tensile strength [37]. They found that it is not easy to achieve a homogeneous distribution of nanoparticles in polymeric matrix since they have a strong tendency to fix agglomerate, especially at higher nanoparticle content. Thus, it is expected to negatively impact the mechanical strength in the PVA/TiO_2_ hydrogel by increasing the TiO^2^ more than the specific nanoparticles’ content. Thus, it is essential to control the method of preparing the polymeric composite to the optimum amount of nanoparticle additive that resulted in the highest mechanical behavior. 

In the case of Fe_2_O_3_ nanoparticles, the addition of small amounts of Fe_2_O_3_ to the PVA gel has a small influence on the mechanical strength; this is due to the small trap of iron oxide nanoparticles in the PVA polymeric chain and inhomogeneous distribution of the nanoparticles within the hydrogel, resulting in more space that is filled with water. Most previous studies have highlighted the addition of iron oxide at high contents, compared with this study. Baqiya et al. concluded that by increasing Fe_2_O_3_ nanoparticles content to around 10%, the space filled and the nanoparticles coincide with the PVA chains, resulting in better crosslinking [39]. The increase in the crystal formation during the physical crosslinking in the PVA/nanoparticles results in the formation of small pore sizes and high mechanical behavior. The PVA hydrogel’s mechanical strength depends on the degree of crystallinity since PVA is a semi-crystalline polymer. The influence of iron oxide on the mechanical behavior of the PVA hydrogel was confirmed by Bannerman, when he compared the elastic modulus for the PVA/iron oxide hydrogel and with the sample after releasing the iron oxide. The removal of iron oxide from the PVA gel sample resulted in a 50% reduction in the elastic modulus of the PVA gel sample. They indicated that the release of iron oxide weakened the material and proved that the material had reduced crosslinking [40].

One of the major factors that improved the compression strength of the PVA/nanoparticles hydrogel composites, is the formation of compact porous structures. Clearly, PVA hydrogels with uniform pore distribution and small pore diameter resulted in high mechanical strength. In this study, PVA/TiO_2_ with 0.1% TiO_2_ has small pores with an average pore size of 20 µm and showed the maximum Young’s modulus (1.0 MPa). PVA/Fe_2_O_3_ gel composites achieved the maximum Young’s modulus (0.6 MPa) at 1.0 wt% Fe_2_O_3_ that has small pore size of 25 µm. However, for other PVA hydrogels with higher pore sizes, lower mechanical strength was observed. Hou et al. related the improvement in the compression strength with the regular pore’s distribution and the formation of small pore size; in their study, the compression strength increased by 72% and the pore size decreased from 15 to 2 µm by increasing the Fe_2_O_3_ from 0 to 10 wt%. The authors tested the effect of the magnetic Nano-hydroxyapatite-coated by ɣ-Fe_2_O_3_ (m-nHAP) in the PVA gels. The compression strength of the PVA gel was 8.4 MPa, whereas the addition of 10% iron oxide to the PVA gel increased the compression strength to 29.6 MPa, and further increasing in the nanoparticles resulted in the reduction in the mechanical strength [20]. Similar results were obtained by Baqiya et al. by adding different wt% of Fe_2_O_3_ to the PVA gel, and the results showed that increasing the iron oxide nanoparticles to the PVA gel increased the mechanical strength by adding 10 wt% Fe_2_O_3_ [39]. It was proposed that there was no significant improvement in the modulus of elasticity, where the modulus of elasticity increased from 0.17 to 0.74 MPa by increasing the Fe_3_O_4_ from 5 to 12.5 wt%.

Several studies demonstrated the role of the pore size of the PVA/TiO_2_ hydrogel composites in enhancing the hydrogel mechanical strength and the size of the pores. Wang et al. figured that with the addition of 60% dimethyl sulfoxide (DMSO) and 0.3 wt%, many pores were observed inside the hydrogel with about 10 μm and the hydrogel strength increased by 12 times with increasing the TiO_2_ content from 0 to 0.3 wt% In comparison, a negative impact on the tensile strength was observed at higher TiO_2_ ranges [19]. The effect of TiO_2_ on the mechanical strength of different hydrogel composites was investigated, and most previous studies agreed with this study. It was clear that the optimum TiO_2_ content that influenced the mechanical strength varied according to the hydrogel type and preparation method. For example, El-Aassar et al. showed that the tensile strength improved by increasing TiO_2_ NPs content to 0.03%. However, the stress increased from 0.7 to 1.0 MPa at 13% strain, and a further increase in the nanoparticle content harmed the mechanical behavior due to agglomeration of TiO_2_ particles that led to early breaking. They studied the effect of TiO_2_ nanoparticles on the mechanical behavior of nanofibers consisting of polyvinyl alcohol (PVA), Pluronic F127 (Plur), and polyethyleneimine (PEI) [41]. Siripatrawan and Kaewklin concluded that the maximum hydrogel strength was obtained by adding 1.0 wt% TiO_2_ to chitosan, and a negative impact was observed at 2.0 wt% TiO_2_.

The maximum tensile strength was 16 MPa compared to the pure chitosan matrix, which achieved around 11 MPa tensile strength [36]. The mechanical characterization of the biopolymer iota-carrageenan (CRG) and PVA hydrogel with different TiO_2_ nanoparticles content (0.25, 0.5, and 0.75%) was compared with the tensile strength and Young’s modulus. Similar behavior was observed where the tensile strength increased with increasing the TiO_2_ by adding 0.25%, and further increasing resulted in a reduction in the mechanical properties of the hydrogel. Compared to pure hydrogel, the tensile strength and Young’s modulus were improved by 20% and 8%, respectively [18]. The Young’s modulus was higher than that achieved in this study, where the PVA polymer was incorporated with other polymers (iota-carrageenan), and the polymer composition was 25% for each type of polymer. This can justify the difference in the amount of the TiO_2_ that influences the mechanical properties compared to this study.

The properties of PVA hydrogels prepared by the cyclic freezing–thawing technique have been examined extensively and depend upon various factors, including PVA concentration, the number of thermal cycles, the type of composite hydrogel, and the reinforcement type and content. The enhancement of the mechanical properties using polymers reinforced by nanoparticles is generally due to several properties of nanoparticles, including their small size and the presence of unpaired atoms; this resulted in a combined potential with the polymer substrate [38,42]. Furthermore, a noticeable enhancement was observed in the porous structure and polymeric network by adding TiO_2_ and Fe_2_O_3_ nanoparticles to PVA gel composites. However, several reinforcement materials for PVA may have a remarkable effect on pore distribution and pore size. Furthermore, some additives had a negative impact on the porosity of the hydrogel. For example, it was reported that the addition of chitosan increased the pore size of the prepared PVA gel, and salecan addition resulted in regular pore distribution and increased the porosity of the hydrogel [5,43]. In addition, the reinforcement of PVA gel with cellulose may not have any effect on the morphology or may have a negative effect on the pore distribution and pore size [42,43].

Additionally, incorporating Cellulose Nano-whisker (CNW) into PVA gel prepared by three F-T cycles resulted in a smaller pore size and lower pore size dispersion for the samples with reinforcement [44]. The improvement in the mechanical strength of hydrogels has been intensively investigated using different reinforcements. Among these, the addition of nanoparticles for hydrogel preparation is one of the most deeply investigated. The mechanical strength of PVA hydrogel is usually characterized using tensile or compression tests. Since the characterization of PVA gel and PVA composite hydrogel depends on several preparation conditions, a wide range of values is obtained for the results of the mechanical behavior. Thus, the best way to investigate the effect of the addition of any reinforcement type or quantity is to compare it with the pure PVA gel prepared under the same conditions. Most previous studies discussed the preparation of PVA gel composites at a small number of freezing–thawing cycles ranging from 2–5 cycles (Table 1). The formulation of the PVA composite involved a combination of diverse natural and synthetic materials, such as cellulose, chitosan, salecan, graphene oxide, and titanium oxide. This synthesis process was conducted under various freezing–thawing conditions. The incorporation of multiple nanomaterials into the PVA gel significantly enhanced its mechanical strength, as evaluated through tests for both tensile strength and compression strength. Hoon et al. [43] investigated the effect of chitosan on the porous structure and mechanical strength of PVA hydrogel. They concluded that by increasing the chitosan content from 2.5 to 10%, the Young’s modulus decreased from 539 MPa to 29 Mpa, indicating a 1755% reduction in the mechanical strength combined with larger pore size by adding chitosan [45]. The chitosan–PVA polymer matrix affects the formation of PVA crystalizes and leads to the formation of less ordered structure hydrogels [46]. It was proposed that the addition of cellulose nanocrystals (CNC) from 10 to 30 g/L increased the viscoelastic properties and the storage modulus from 11 Pa to 391 Pa. They concluded that the network structure between CNC particles and the PVA polymer chains resulted in the enhancement of viscoelastic behavior [47]. Therefore, choosing the suitable type and content of the material added to the PVA gel ensures the interaction with the PVA gel to improve the porous structure and mechanical strength.

The preparation of PVA composite was obtained using several natural and synthetic materials, including cellulose, chitosan, salecan graphene oxide, and titanium oxide, at several F-T cycles (Table 1). The addition of several nanomaterials to PVA gel improved the mechanical strength tested in terms of tensile strength and compression strength. The enhancement of the mechanical properties using polymers reinforced by nanoparticles is generally due to several properties of nanoparticles, including their small size and the presence of unpaired atoms; this results in a combined potential with the polymeric substrate [38,42]. This study marked a noticeable enhancement in the porous structure and mechanical strength by adding TiO_2_ and Fe_2_O_3_ nanoparticles to the PVA gel composites. Similarly, other materials resulted in such an improvement in the mechanical behavior of hydrogels. For example, incorporating Cellulose Nano-whisker (CNW) into PVA gel prepared by three F-T cycles resulted in smaller pores and lower pore size dispersion for the samples with reinforcement [44]. It was proposed that the addition of cellulose nanocrystals (CNC) from 10 to 30 g/L increased the viscoelastic properties and the storage modulus from 11 Pa to 391 Pa. They concluded that the network structure between CNC particles and the PVA polymer chains resulted in the enhancement of viscoelastic behavior [47]. Although many other fillers had a similarly remarkable effect on the reinforcement of the PVA hydrogels, other additives had no impact or negatively affected the network and structure of the hydrogel composites, and hence the mechanical strength. The addition of chitosan from 0.25 to 1.0 wt% resulted in a reduction in the mechanical strength and increased the pore size of the PVA hydrogel composites. They concluded that by increasing the chitosan content from 2.5 to 10%, the Young’s modulus decreased from 539 MPa to 29 Mpa, indicating a 1755 % reduction in the mechanical strength combined with larger pore size by adding chitosan [43]. The chitosan–PVA polymer matrix affects the formation of PVA crystalizes and leads to the formation of less ordered structure hydrogels [46]. The addition of salecan to the PVA hydrogel also resulted in a reduction in mechanical strength. The pure PVA hydrogel yielded the highest compressive strength and modulus, while the values of the hybrid hydrogels decreased as the proportion of Sal/PVA increased. For example, the compressive modulus of the hydrogels declined considerably with the increase in salecan content from 145 kPa (pure PVA) to 23 kPa (S50P50). This is explained by the formation of stronger interaction and crosslinking in PVA hydrogel itself than that between PVA and salecan, which was not strong compared to the crosslink between PVA itself [5]. Therefore, it is important to choose the suitable type and content of the material that will be added to the PVA gel to ensure the interaction with the PVA gel in order to improve the porous structure and the mechanical strength.

### 4.3. Performance of PVA/Nanoparticles Hydrogel in Bioreactor

The industrial implementation of an immobilization matrix will require high material stability to withstand the shear stresses and abrasion in the bioreactor environments, both for long-term operation and biocatalyst reuse. Among several PVA/nanoparticles composites developed in this study, the PVA/TiO_2_ with 0.1 wt% achieved the most remarkable improvement in mechanical strength. Additionally, the durability test showed that the reinforcement of PVA hydrogel using TiO_2_ nanoparticles improved hydrogel strength by more than 50%. The pore structure of the immobilization matrix is an essential factor in biomass immobilization using the entrapment method. The choice of PVA hydrogel for biomass immobilization is due to its porous structure and hence the excellent performance in biomass housing to form in its pores for growth and cultivation [50]. Furthermore, the pores generate the possibility of efficient diffusion of organic particulates and soluble pollutants into the immobilization matrix [51]. According to the SEM analysis, PVA/TiO_2_ hydrogel is a homogenous porous material suitable for biomass immobilization. Thus, it was chosen and tested for biomass immobilization and biological treatment of GTL process water. Immobilized bacteria in PVA/TiO_2_ showed high biodegradability due to the distribution of the regular pores that allowed biomass immobilization in cross-linked PVA hydrogel composites and enhanced the diffusion of the organic pollutants through the pores in the PVA gel.

## 5. Conclusions

The preparation of PVA nanocomposites for biomass immobilization is a good alternative to conventional PVA gel matrices. The reinforcement of PVA hydrogel by Fe_2_O_3_ and TiO_2_ influenced the morphological structure and mechanical strength of the PVA nanocomposite hydrogel. The morphological analysis showed that the reinforcement of PVA gel with Fe_2_O_3_ and TiO_2_ nanoparticles resulted in compact nanocomposite hydrogel with regular pore distribution and tiny pore diameter due to the formation of bonds between nanoparticles and hydrogel that caused more interaction within the polymeric matrix. Furthermore, the hydrogel’s mechanical strength and elasticity depend on the type and content of the nanoparticles. The most remarkable improvement in the mechanical strength of the PVA/nanoparticles composites was obtained by incorporating 0.1 wt% TiO_2_ and 1.0 wt% Fe_2_O_3_ nanoparticles. However, TiO_2_ has more influence on the morphology and mechanical strength than iron oxide since approximately 900% improvement in the Young’s modulus resulted from optimization of the content of the TiO_2_ in the PVA hydrogel.

The PVA/TiO_2_ matrix was used for biomass immobilization and compared with pure PVA gel to treat the GTL process water. The addition of nanoparticles to the PVA matrix improved the mechanical strength but did not affect the biodegradation performance. The current study illustrated that the developed PVA/nanoparticles hydrogel is a suitable immobilization matrix and can replace the conventional PVA hydrogel in biomass immobilization and the area of biological wastewater treatment.

## Figures and Tables

**Table 1:** Examples of the mechanical strength and morphology of PVA gel nanocomposites.

Additive	Composition	Preparation Conditions	Morphology	Mechanical Strength	Ref.
chitosan minocycline	2.5–10% (*w*/*v*) PVA 0.3–1.12% (*w*/*v*) Chitosan minocycline (0 or 0.25%)	Freezing at −20 °C for 18 h Thawing at 25 °C for 6 h Three F-T cycles	The higher the chitosan, the greater the porous size of the hydrogel	The Young’s modulus using tensile strength for pure PVA was 539 Mpa and decreased to 29 Mpa, indicating a 1755% reduction in the improvement.	[43]
Salecan	10% (*w*/*v*) PVA. 2% *w*/*v* salecan	Freezing at −20 °C for 18 h. Thawing at 25 °C for 6 h Three F-T cycles	Interconnected porous structure with regular pore distribution	The compressive modulus of the hydrogels decreased considerably with the decrease of salecan content from 145 kPa (pure PVA) to 23 kPa (PVA 5% and salecan 1%).	[5]
Cellulose nanowhisker (CNW)	10%(*w*/*v*) PVA 1, 3, 5, and 7 wt% of CNW	Freezing at −18 °C for 1 h Thawing at 25 °C for 1 h Three F-T cycles	Smaller pore size and the lower pore size dispersion for the samples with reinforcement	Young’s modulus increased from 0.8 to 1.1. MPa by adding 3% CNW to the PVA gel	[44]
Nanocrystalline cellulose (CNC)	5, 7.5, 10%(*w*/*v*) PVA 1 wt% CNC	Freezing at −20 °C for 24 h Thawing at 25 °C for 2 h 3 and 5 F-T cycles	The addition of CNC does not have any effect on the morphology of the dried gels	The compression stress was increased by 24% by adding 1 CNC to the PVA gel (10 *w*/*v*) for 3 F-T cycles and increased by 15% for 5 F-T cycles.	[48]
Graphene oxide (GO). boron-cross-linked(B-GO/PVA) hydrogels prepared by F-T method and immersed in boric acid.	10% (*w*/*v*) PVA 0–0.2% graphene oxide (GO)	Freezing at −20 °C for 8 h Thawing at ambient temperature for 2 h 5 F-T cycles	-	Compared to B-PVA hydrogel, the B-PVA/GO 0.1 wt% the tensile strength increased 144% (0.609 MPa), and the compression and shear strength increased by 26% and 35% (0.1 MPa and 0.201 MPa).	[49]
Cellulose nanocrystals (CNC)	15% (*w*/*v*) PVA 0.75, 1.5, and 3% CNC	2–3 F-T cycles	-	The maximum compressive strength at 60% strain of 53 kPa was obtained from the hydrogels with 1 wt% CNCs, which was 303% higher than that of the neat PVA hydrogel (17.5 kPa).	[27]
Titanium oxide (TiO_2_) NP	15% (*w*/*v*) PVA 0.3 to 0.6 TiO_2_ wt% 2 wt% Na_2_CO_3_ and DMOS 60%	Freezing at −18 °C for 12 h Thawing at 15 °C for 4 h 3 F-T cycles	The composite hydrogel has a porous structure with pore dimeter of about 10 μm.	The optimum material ratio and preparation conditions resulted in tensile strength of the prepared composites of 14.3 MPa by using 0.3 TiO_2_ compared to pure PVA that achieved only 1.0 MPa.	[19]
magnetic nano-hydroxyapatite-coated ɣ-Fe_2_O_3_ (m-nHAP)	10 wt% PVA and m-nHAP of 10, 20, 50 and 80%.	6 F-T cycles	The average pore size of the nanocomposite hydrogels has a minimum of 1.6 ± 0.3 μm at 10 wt% of m-nHAP.	Compressive strength reached the maximum value of 29.6 ± 6.5 MPa at 10 wt% of m-nHAP.	[20] This study
Titanium oxide (TiO_2_) NP	10% (*w*/*v*) PVA 0–1.0% TiO_2_	Freezing at −20 °C for 24 h Thawing at 20 °C for 5 h 4 F-T cycles	An improvement in the pores and network structure with highest regular and small pore size at 0.1 TiO_2_ wt%	Young’s modulus increased from 0.011 to 1.0 MPa by adding 0.1 TiO_2_ nanoparticles to the hydrogel.	This study
Iron Oxide (Fe_2_O_3_) NP	10% (*w*/*v*) PVA 0–1.0% Fe_2_O_3_	Freezing at −20 °C for 24 h Thawing at 20 °C for 5 h 4 F-T cycles	A remarkable improvement in the porous structure especially by adding 0.04 and 0.1 wt%.	Young’s modulus increased from 0.011 to 0.6 MPa by adding 1.0 Fe_2_O_3_ nanoparticles to the hydrogel.	This study

## Data Availability

The data presented in this study are available upon request from the corresponding author. The data are not publicly available due to ethical considerations, confidentiality agreements with participants, and the necessity to protect sensitive information and maintain participant privacy.
